# Child Mortality Estimation: Methods Used to Adjust for Bias due to AIDS in Estimating Trends in Under-Five Mortality

**DOI:** 10.1371/journal.pmed.1001298

**Published:** 2012-08-28

**Authors:** Neff Walker, Kenneth Hill, Fengmin Zhao

**Affiliations:** 1 Bloomberg School of Public Health, Johns Hopkins University, Baltimore, Maryland, United States of America; 2 Harvard School of Public Health, Harvard University, Cambridge, Massachusetts, United States of America; Umeå Centre for Global Health Research, Umeå University, Sweden

## Abstract

In most low- and middle-income countries, child mortality is estimated from data provided by mothers concerning the survival of their children using methods that assume no correlation between the mortality risks of the mothers and those of their children. This assumption is not valid for populations with generalized HIV epidemics, however, and in this review, we show how the United Nations Inter-agency Group for Child Mortality Estimation (UN IGME) uses a cohort component projection model to correct for AIDS-related biases in the data used to estimate trends in under-five mortality. In this model, births in a given year are identified as occurring to HIV-positive or HIV-negative mothers, the lives of the infants and mothers are projected forward using survivorship probabilities to estimate survivors at the time of a given survey, and the extent to which excess mortality of children goes unreported because of the deaths of HIV-infected mothers prior to the survey is calculated. Estimates from the survey for past periods can then be adjusted for the estimated bias. The extent of the AIDS-related bias depends crucially on the dynamics of the HIV epidemic, on the length of time before the survey that the estimates are made for, and on the underlying non-AIDS child mortality. This simple methodology (which does not take into account the use of effective antiretroviral interventions) gives results qualitatively similar to those of other studies.

## Abstract 2

Neff Walker and colleagues show how the United Nations Inter-agency Group for Child Mortality Estimation use a model to correct for AIDS-related biases in the data used to estimate trends in under-five mortality.

## Introduction

HIV/AIDS is different from many causes of mortality in that people are primarily infected and subsequently die during their peak reproductive period. In countries with generalized HIV epidemics, taken here as having a prevalence that reaches 5% of the adult population, HIV incidence peaks among women in their mid-20s and among men approximately five years older. Given an average survival time of around ten years in the absence of antiretroviral therapy (ART), this means that, prior to the widespread use of ART, AIDS mortality in countries with generalized HIV epidemics peaked around age 30–35 years in women and around five years older for men [1]. For these countries, all of which are in sub-Saharan Africa, AIDS is the leading cause of mortality among adults of reproductive age (15–49 years old) [2]. A study conducted early in the epidemic calculated that in the countries of southern Africa with HIV prevalence over 10% [3], almost half of all adults in the 15-year-old cohort would die due to AIDS, and population growth would cease [4].

A second unique feature of HIV/AIDS is the transmission of HIV from infected mothers to their children. Prior to widespread use of ART, infected children were almost certain to die in childhood, thus introducing a strong correlation between mortality risks of mothers and their children.

In this review, we discuss how this combination of high prevalence, high mortality, and a correlation between the mortality risks of mothers and their children has a major impact on the data used to estimate the under-five mortality rate (the probability of dying between birth and age five years, also denoted in the literature as U5MR and _5_ *q*_0_). We review the approach currently being used by the United Nations Inter-agency Group for Child Mortality Estimation (UN IGME) to adjust for bias due to AIDS in child mortality estimation (CME) in countries where the prevalence of HIV/AIDS has reached 5% or above in the adult population. We discuss the impact this adjustment has on estimates of under-five mortality and conclude by discussing future plans within UN IGME to expand this procedure and to improve the accuracy of the adjustment for the estimation of trends in under-five mortality in countries with a significant HIV epidemic.

## Why Does AIDS Mortality Bias Child Mortality Estimates?

Estimates of child mortality for populations lacking accurate registration of births and deaths (in practice, most low- and middle-income countries) are derived almost entirely from reports of mothers of reproductive age about the survival of their children. The most common approach to the collection of the data needed for CME—the collection method used in all surveys by the Demographic and Health Surveys (DHS) program and in some surveys by the Multiple Indicator Cluster Surveys program—involves taking full birth histories. In such histories, each woman aged 15 to 49 years at the time of the survey is asked for the date of birth of each live-born child she has had, and, if the child has died, the age at death. Child mortality is directly estimated from the information on births and deaths for periods up to 25 years before the survey. The births on which data are collected must be representative of all the births in the population for the entire period to allow valid estimates of population-level child mortality to be made. It is unlikely that this condition is ever perfectly met: births to mothers who have died or migrated out of the population will not be reported. Moreover, for periods long before the survey, births to older mothers will not be represented because these mothers will have been age 50 or over at the time of the survey and therefore not included. It is normally assumed that any bias introduced by lack of representativeness is small, but this will not be the case in populations substantially affected by HIV, where HIV-positive children will be more likely to die than other children, and their deaths will be less likely to be reported since their mothers will have also been more likely to die. Direct child mortality estimates will thus be biased downwards in regions affected by a generalized HIV epidemic [5]–[8].

The second most common approach to the collection of data for CME involves collecting summary birth histories. Many developing country population censuses and household surveys, as well as most surveys by the Multiple Indicator Cluster Surveys program, collect summary birth histories. Each woman 15 to 49 years old at the time of the survey is asked how many live-born children she has had and how many of them are still alive. Indirect estimates of child mortality are then derived covering the ten- to 15-year period before the survey from proportions dead of children ever born classified by five-year age groups of mothers through the use of fertility and mortality models [5]. As with full birth histories, mothers of high-risk HIV-infected children are less likely to report because of their own HIV-related mortality, and, as a consequence, child mortality estimates are again biased downwards.

Although migration and selective nonresponse for other reasons may introduce bias, the greatest threat to child mortality estimates based on reports of women about the survival or death of their children currently arises from generalized HIV/AIDS epidemics. Vertical transmission of HIV from mother to child during pregnancy and delivery, and through breastfeeding in the first few months of life, increases the risk that the child will be HIV-positive by as much as 35% in the absence of ART, and over 60% of HIV-positive children will die before their fifth birthday in the absence of ART treatment [9]–[11]. Since the mothers also suffer elevated mortality risks, the deaths of many of these HIV-positive children, particularly those born five years or more before interview, will not be reported. Overall child mortality will therefore be underestimated, whether using direct or indirect CME methods.

## How Can the Effects of AIDS Mortality on Direct CME Be Calculated?

To our knowledge, only one analysis of the magnitude of bias in direct child mortality estimates due to AIDS mortality has been carried out using real data rather than simulations. Hallett et al. [8] used data from a prospective open cohort in Manicaland, Zimbabwe, to measure the bias introduced by deaths of HIV-positive mothers. The cohort was interviewed between July 1998 and February 2000, with follow-up interviews at three and five years; at each interview round, child deaths since the previous round were recorded. From 1998 to 2005, HIV prevalence in the study population fell from 22% to 18%. In the final interview round in 2005, a full birth history was collected from the surviving women, and U5MR was estimated for the period 1998 to 2005, a seven-year period as opposed to the five-year periods usually used for DHS estimates. The direct estimates were then compared to true values, adding back the child mortality experience of women who had died before 2005. The bias, calculated as the estimates from reports by surviving mothers divided by the estimates for all mothers, was 6.7% for the infant mortality rate (the probability of death before age one year) and 9.8% for the U5MR. The analysis indicated that bias in direct estimates increases with the duration of the epidemic and with the time before survey of the estimate, but decreases as the level of background non-HIV child mortality increases. Hallett et al. also developed a model of bias, which they applied to Zimbabwe and six other countries with moderate or high HIV prevalence for the period 1980 to 2015 using Joint United Nations Programme on HIV/AIDS (UNAIDS) prevalence data and DHS estimates for pre-epidemic periods. Thus, the Hallett et al. analysis only directly estimates bias for the five-year period before a survey; estimates for earlier periods are model-based.

### The UN IGME Approach to Bias Adjustment in Populations with a Generalized HIV Epidemic

Based on the findings of Hallett et al. [8], UN IGME [12] recently implemented an adjustment approach for use in countries where prevalence of HIV/AIDS has reached 5% or above in the adult population (ages 15–49 years). Because precise estimation of the bias in reported U5MR due to HIV would require a great deal of information about the HIV epidemic that is not typically available (for example, details on the distribution of births to HIV-positive women by the duration of infection, vertical transmission rates, and survival times of both mothers and children from the time of the birth), UN IGME has adopted a number of simplifications.

The UN IGME approach uses a simple cohort component projection model that is implemented in a customized Excel workbook and that starts with the latest projection of a national population and its HIV epidemic from UNAIDS [3]. The HIV/AIDS projections are made using Spectrum population projection software [11] and combine data on HIV prevalence and a resulting incidence curve with assumptions about many factors, such as survival time with and without treatment and age-and sex-specific prevalence, to produce an estimate of the complete course of the epidemic from its start to the current date. The Spectrum output provides the annual number of births, typically from 1970 onwards, the number of women each year in need of prevention of mother-to-child transmission (taken as a proxy for the number of births to HIV-positive women), and the number of HIV-positive infants. The input data for the UN IGME model are taken directly from the Spectrum output and thus take into account the fertility-reducing effects of HIV and the estimated transmission of HIV from mother to child. The Spectrum model also takes into account breastfeeding patterns and the impact of various interventions to prevent mother-to-child transmission in its estimation of the number of children infected with HIV.

For each year, the births are divided into three streams: HIV-negative births to HIV-negative mothers, HIV-negative births to HIV-positive mothers, and HIV-positive births to HIV-positive mothers (no distinction is made between children infected at or before birth and those infected after birth). For births in each year, deaths under age five in the subsequent five years are calculated for each stream. For both categories of HIV-negative births, risks of dying in each year from birth to age five are obtained from a model life table (the Coale and Demeny “West” family [13]) with a U5MR approximating a best guess of the U5MR in the HIV-negative population, referred to as the background U5MR. The simplifying (and somewhat unrealistic) assumption is made that the mortality risks of HIV-negative children are the same regardless of the HIV status of the mother. For HIV-positive births, the model uses a mortality schedule derived by averaging results from cohort studies [9]–[11], with a probability of dying by age five of 62.5%, to provide risks of dying in each year from birth to age five; it is assumed that ART treatment had no effect on these risks until after 2007.

From this series of steps, the model provides estimates of “true” births and under-five deaths for each calendar year. The next step is to estimate how many of these births and under-five deaths will go unreported at a particular survey because of the deaths of mothers. To do this, the UN IGME model first assumes that HIV-negative women have negligible mortality risks over the short time frame involved, so all their births and under-five deaths are reported. It then makes the further simplifying assumption that births to HIV-positive women occur to women four years after infection (four years was chosen on the grounds that births to HIV-positive women are skewed towards the beginning, rather than the end, of their infected life). A survival curve from first infection, again derived from cohort studies [9]–[11], with a median survival time of about 9.5 years, is used to create a survival curve from four years after infection, from which the probabilities of surviving from a particular year to the year of a given survey (assumed to be at the end of a year) is obtained. These curves are then used to estimate the proportion of the births and child deaths (whether HIV-negative or HIV-positive) of HIV-positive mothers that would have been reported by the survey if all of the mothers had survived to the time of the survey.

For each five-year period before a survey, the “true” and the “reported” births and under-five deaths are summed; the five-year periods used are 1–5, 6–10, and 11–15 years before the survey to reduce the impact of the “displacement effect” often found in DHS datasets, a tendency to shift births backwards in time from four years before the survey to five years before [14]. The estimated bias for each period is calculated as 1.0 minus the “reported” ratio of under-five deaths to births (reported by women still alive at the survey) to the corresponding ratio that would have been observed had none of the mothers died. Finally, survey estimates of under-five mortality are adjusted by dividing by 1.0 minus the estimated bias for each period. Box 1 provides a simplified example of this process.

**Box 1. An Example of How the UN IGME Cohort Component Projection Model Works for a Survey Conducted in 2005**

- We assume the survey was conducted at the end of 2005 and consider births that occurred in 1995. Spectrum provides the number of such births, the number of women in 1995 in need of services to prevent mother-to-child transmission (a proxy for the number of births to HIV-positive women), and the HIV status of infants that year (assumed equal to incidence among that year's births).
- From these figures, we derive the number of births to HIV-negative and HIV-positive women.
- Births to HIV-positive women are then subdivided as per Spectrum output into those that will, and those that will not, become infected with HIV, reflecting breastfeeding patterns and prevention of mother-to-child transmission interventions.
- We estimate an appropriate U5MR for HIV-negative births (largely from estimates prior to the epidemic), let's say, 150 per 1,000 live births. Under-five deaths will occur in the period 1995–2000, by which time all the surviving children will have reached age five. All the births and under-five deaths to HIV-negative women will be reported, but few of the births and deaths (regardless of the serostatus of the child) to HIV-positive mothers will be reported (the ten-year survival probability from four years after infection in our model is only 14%).
- This process is repeated for all birth cohorts from 1990 to 2005. True births and under-five deaths, and reported births and under-five deaths, are summed for the periods 2000–2004, 1995–1999, and 1990–1994 (corresponding to 1–5, 6–10, and 11–15 years before the survey, and starting at one year before the survey to minimize the birth displacement found in many DHS surveys). For each period the ratio of reported proportion dead to true proportion dead is calculated.
- Finally, survey estimates of U5MR for each period are adjusted by dividing the observed values by 1.0 minus the ratio just calculated.

Once the adjusted estimates of U5MR have been made, they are used as the inputs into the UN IGME fitting approach that is used to develop the trends and current point estimates of U5MR for all low- and middle-income countries [15]. The fitting approach is described in detail in another paper in the 2012 *PLOS Medicine* Collection “Child Mortality Estimation Methods” [14]; the adjusted and unadjusted datasets and country-specific data fits are all available in the CME Info database (http://www.childmortality.org). Importantly, the fitting approach used for countries with HIV epidemics is different from that used for those without, and can be summarized as follows. First, the bias-adjusted U5MR estimates are calculated from each DHS survey. Second, HIV deaths for each period as estimated by UNAIDS are subtracted to obtain non-HIV U5MR estimates. Third, a loess curve is fitted to the non-HIV U5MR estimates to obtain a smooth time sequence. Finally, the HIV deaths by period are added back to obtain the final adjusted U5MR series [14].

**Figure 1:**
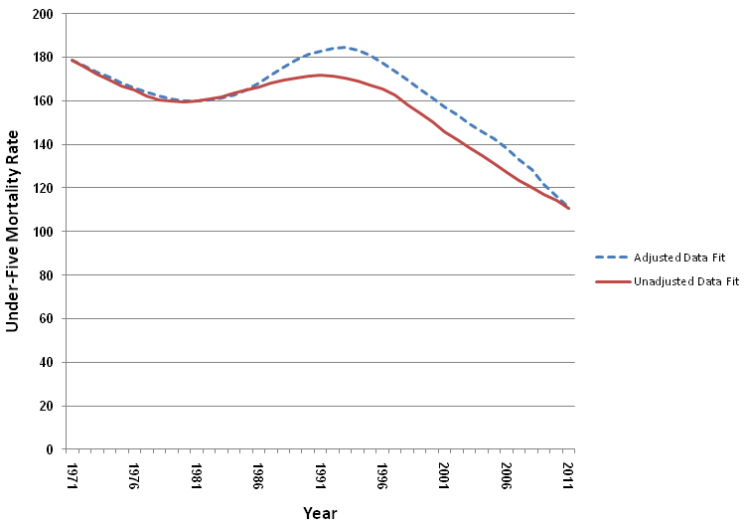
Best fitting trends by year in U5MR using adjusted direct datasets compared to unadjusted datasets.

A detailed example of the adjustment procedure for Zambia in 2007 is available in Protocol S1. Figure 1 shows the different best fitting trends in under-five mortality for Zambia, using the unadjusted and adjusted datasets. Zambia is a good example, as there have been four DHS surveys that used direct methods for childhood mortality measurement (1992, 1997, 2002, and 2007), and the estimates of adult HIV prevalence for Zambia have been above 10% since the mid-1990s [3]. As can be seen in Figure 1, the deviation between the unadjusted and adjusted curves starts in the mid-1980s in Zambia, the period after the presumed start of the HIV/AIDS epidemic in the country, when AIDS mortality becomes an issue. Prior to that period, the curves are identical, as there is no adjustment for AIDS mortality. The two curves converge at the end, as there is only one recent data point (from the 2007 DHS survey) and the adjustment for the recent recall period is small.

## How Big Should the Bias Adjustment Be?

Table 1, which was prepared using the UN IGME model described above, shows estimates of bias for six countries in sub-Saharan Africa based on DHS data collected around the middle of the last decade, before ART was widely available, for time periods 1–5, 6–10, and 11–15 years before each survey. It is clear from this table that the extent of bias depends on the HIV prevalence and its past trajectory, the level of background U5MR, and the time period before the survey to which the estimate refers. The Hallett et al. analysis [8], which is based on actual data, reaches the same conclusions. Although Table 1 provides a general guide to the size of bias adjustment likely to be needed, it clearly indicates that it is not possible to provide a simple way to assess the magnitude of bias. It is also important to remember in interpreting these results that bias is a function of non-HIV-related child mortality, which is not easy to estimate, and HIV prevalence, which is usually estimated with error. Notably, however, Table 1 shows that bias is highest for the period 6–10 years before the survey and is 10% or more if the HIV prevalence exceeds 5%. The highest bias (26%) was estimated for Zimbabwe, which has a high HIV prevalence and moderate background mortality.

**Table 1:** Estimates of bias for estimates of U5MR for periods 1–5, 6–10, and 11–15 years before each survey: selected sub-Saharan African countries.

Country	DHS Year	Approximate HIV Prevalence in 2005 (Percent)	Assumed Background U5MR	Estimated Bias by Period before Survey (Percent)			Hallett et al. [8] Estimate 2005–2009
				1–5 Years	6–10 Years	11–15 Years	
Côte d'Ivoire	2005	4.6	125	3.0	4.5	1.9	<2
Kenya	2003	7.1	75	6.7	9.8	3.0	N/A
Lesotho	2004	23.4	75	11.0	11.1	1.1	8
Namibia	2006–2007	15.3	50	11.5	17.1	6.6	6
Zambia	2007	15.0	150	5.3	10.8	9.1	4
Zimbabwe	2005	18.0	75	14.8	26.5	19.1	13

N/A, not available.

## Application of Methods to Adjust for Bias due to AIDS

The 2010 round of estimates from UN IGME [15] was the first set of estimates that used the systematic approach described above to adjust for known biases due to AIDS mortality in the direct methods of measuring U5MR. In all, adjustments were made for 17 countries and 49 surveys. All the datasets for U5MR, including the adjusted datasets where appropriate, are available in the CME Info database (http://www.childmortality.org). This publicly available database offers the opportunity for national programs and researchers to access the datasets and to try different fitting approaches online.

While we believe the use of the adjusted datasets has improved the estimates of U5MR in countries where there is a generalized HIV epidemic, the methodology makes a number of simplifying assumptions. In particular, it makes the following key assumptions. (i) It assumes that HIV-negative children of HIV-positive mothers experience the same background mortality as children of HIV-negative mothers. This is unlikely to be correct; there is evidence that such children also suffer excess mortality, though not from HIV. To the extent that this is the case, the methodology will underestimate bias. (ii) The model does not distinguish the survival prospects of those infected before or at birth from those infected through breastfeeding. (iii) The methodology assumes that births to HIV-positive women all occur four years after infection, with correspondingly shortened survival times for the mothers. We have no insight as to which direction this simplification might affect estimates of bias. (iv) Related to assumption iii, the methodology does not account adequately for the early dynamics of an epidemic, when a large proportion of HIV-positive mothers are at an early stage of infection. This simplification is likely to overestimate bias for the first few years of an epidemic. (v) The methodology assumes that HIV-negative women experience no mortality. This is the same assumption as the “no selection bias” assumption made in standard analyses of birth histories. Our hypothesis is that this assumption may lead to a slight overrepresentation of HIV-negative women in the estimates, and therefore a slight overestimate of bias. (vi) The methodology assumes we can adequately estimate background non-AIDS U5MR. (vii) Finally, the current model makes no adjustment for ART. As therapies become more widespread, the excess U5MR of children of HIV-positive women will decline, and the mortality of their mothers will also decline, so any bias will decline. The effect of treatment on bias in surveys conducted prior to 2008 should be small, but going forward we will need a methodology that takes into account the dynamics of infection and treatment.

Table 1 compares our estimates of bias with the estimates previously modeled by Hallett et al. [8]. In all cases, our biases are larger, often by a substantial margin (double in the case of Namibia). Importantly, however, the rank ordering of countries by bias is almost identical. One reason our estimates are higher may be that our time periods are for the early 2000s, whereas Hallett et al.'s are for the late 2000s; another reason may be that we assume lower background non-HIV child mortality, but this could explain only a small proportion of the differences. The case of Namibia is particularly marked: the country has high HIV prevalence and low background mortality, yet relatively small bias as estimated by Hallett et al. [8]. Our estimate of bias for this country may be considerably higher than that provided by the Hallett et al. model because of assumption iv above—the HIV epidemic in Namibia is younger than that in some other countries considered in Table 1.

Given the large and continuing uncertainty about the dynamics of the HIV epidemic, we contend that simplifying assumptions are justified for the purpose at hand. Ultimately, of course, rather than complex models with many parameters, it is desirable to have more empirical estimates of bias based upon surveillance sites, such as those of Hallett et al. [8]. It should also be borne in mind that the adjustment for bias in direct CMEs is only one part of the overall adjustment. As explained earlier, once a direct survey-based U5MR estimate has been adjusted for HIV bias, it is adjusted again to approximate an AIDS-free value on the basis of the UNAIDS-estimated number of AIDS deaths of children under five. The loess curve is then fitted to all the available estimates of AIDS-free U5MR, including those for time periods prior to the epidemic. Under-five AIDS deaths are then added back to the AIDS-free trajectory of U5MR to get final estimates. There is substantial variation in estimates of under-five AIDS deaths for a given year from one UNAIDS revision to the next; the adjustment for birth history bias, therefore, is not the only source of substantial uncertainty in the final estimates.

## Future Prospects

We do not regard the current UN IGME approach as a “finished product.” Rather, it is a work in progress. In particular, bearing in mind the fact that this approach makes no allowance for the evolution of the AIDS epidemic (see assumptions iv and vii above), we plan to add refinements that will improve the adjustment methods. One major change we are currently working on is to switch to using estimates of incidence cohorts instead of prevalence cohorts from the UNAIDS models of HIV/AIDS. Beginning with the 2010 round of estimates, the Spectrum package can now produce a historical trend of incident cohorts for mothers and children [11]. This output will allow for a better estimate of probability of dying for both mothers and children and should improve the accuracy of the adjustment approach.

The use of incident cohort outputs from Spectrum will also allow direct adjustment for the impact of prevention of mother-to-child transmission programs, and ART for mothers and children. This is important because ART use to prevent mother-to-child transmission and to extend survival times will have a quick effect on reducing bias for the most recent time period before a survey, though bias for past time periods will persist for a decade or more after effective therapy is introduced because infected mothers will have already died. In the current methods we have maternal and child deaths by time (all adjusted for prevention of mother-to-child transmission programs and treatment), but then have to estimate the distribution of those deaths into mother and child pairs. Using the new outcomes from Spectrum, we will be able to make the adjustment without this step.

UN IGME is also investigating the use of an adjustment technique that could be applied to indirect estimates of under-five mortality based on summary birth histories. Such a technique is not currently available for rapidly changing epidemics, but fortunately most of the countries with an adult HIV prevalence greater than 5% conduct surveys with full birth histories with reasonable frequency. Indirect estimates of under-five mortality based on summary birth history data are based on assumptions that include the major one underlying direct estimation from full birth histories (low correlation between probability of death of the mother and child). To date, there have been at least two studies that have used simulations to estimate the impact that various levels of adult HIV prevalence would have on indirect estimates of under-five mortality [7],[16]. Both analyses suggest that at higher prevalence levels and with reports from older mothers, the bias could be considerable. Importantly, however, the correction approach for the bias developed by Ward and Zaba is based on the assumption that the epidemic is stable [7], that is, it assumes that HIV prevalence is not changing over time. Clearly, this assumption is not appropriate for most if not all of the HIV epidemics in countries in sub-Saharan Africa. UN IGME and its Technical Advisory Group are therefore working on ways to build on this model in order to develop adjustment factors for the AIDS-related bias in surveys that have only indirect measures of child mortality.

**Key Points**

- The HIV epidemic invalidates a key assumption of all approaches to estimating child mortality from reports of mothers concerning the survival of their children, namely, that there is no selection bias operating on reports of mothers that is related to the survival of their children.
- UN IGME has developed a simple cohort component projection model to quantify the magnitude of the HIV-related bias affecting direct child mortality estimates from surveys by Demographic and Health Surveys in sub-Saharan Africa.
- The model indicates that the bias can be substantial, exceeding 25% for the period six to ten years before the survey, in settings with high HIV prevalence and low background mortality, such as Zimbabwe.
- Such biases need to be taken into account when fitting smoothed trends to survey data over time and are included in the latest estimates of child mortality produced by UN IGME for countries that have had HIV prevalence of 5% or higher.
- Importantly, the simple model reviewed here includes many simplifying assumptions that may affect the accuracy of its predictions; in addition, it needs further refinement to adjust for the effect of ART on the evolution of HIV epidemics.

## Supporting Information

Protocol S1 **Spreadsheet to estimate AIDS bias in full birth history estimates of child mortality: example application to Zambia 2007.**
(XLS)

## Abbreviations

ART: antiretroviral therapy
CME: child mortality estimation
DHS: Demographic and Health Surveys
U5MR: under-five mortality rate
UN IGME: United Nations Inter-agency Group for Child Mortality Estimation
UNAIDS: Joint United Nations Programme on HIV/AIDS
